# Oncologists’ palliative care referral behaviour: testing utility of social exchange theory as an explanatory framework

**DOI:** 10.1186/s12904-024-01517-0

**Published:** 2024-07-24

**Authors:** Naveen Salins, Sean Hughes, Nancy Preston

**Affiliations:** 1 Department of Palliative Medicine and Supportive Care, Kasturba Medical College, Manipal Academy of Higher Education, Manipal, Karnataka 576104 India; 2 Division of Health Research, Health Innovation One, Sir John Fisher Drive, Lancaster University, Lancaster, LA1 4AT United Kingdom

**Keywords:** Oncologists, Palliative care, Referral, Behaviour, Social exchange theory

## Abstract

**Background:**

Adults and children with cancer are referred to palliative care infrequently or late. Oncologists often gatekeep these referrals. Social exchange theory is used to explain physician referral behaviour in various clinical settings. Its utility in a cancer palliative care setting is not known.

**Methods:**

We used Karl Popper’s hypothetico-deductive approach to test the hypothesis. The hypothesis was that social exchange theory is a helpful framework for explaining oncologists’ palliative care referral behaviour in a cancer setting. The utility of the theoretical framework was tested against the empiric findings of a systematic review and original research.

**Results:**

Most components of social exchange theory known to explain physician referral behaviour like beliefs about the provider or service, emotions triggered during the professional engagement, its symbolism and stigma, the complexity of the referral task, efforts needed to achieve it, its cost, benefit, and value were similar in a cancer setting. Empirical findings suggest that oncologists provided strategies and solutions to better palliative care integration instead of comparing their existing engagement with potential alternatives and choosing them.

**Conclusion:**

Social exchange theory was found to be helpful in explaining oncologists’ palliative care referral behaviour. To further develop the social exchange theory based on the data used to test it, it is recommended to include feedback and solutions as a component of the theoretical framework alongside a comparison level for alternatives.

## Introduction

People of all ages with cancer worldwide are not referred to palliative care as often as they should be, and the referrals that do occur happen very late in the patient’s life [1–6]. This problem is even more pronounced in low-middle-income countries [7–12]. Due to the higher cure potential in a paediatric oncology setting, many children with cancer only receive palliative care in the very last days of their lives [2, 4, 13, 14]. A systematic review showed that, internationally, the median time between palliative care referral and death is only 19 days [15]. Many children with cancer who need palliative care don’t have access to it [16, 17]. Patients often continue receiving cancer treatments until the end, which can impede symptom management and end-of-life care. [4, 5, 14, 18]. Delayed palliative care referrals often hinder communication and shared decision-making processes [3, 19, 20] and may also lead to unnecessary invasive medical interventions at the end of life [5, 21] and increased in-hospital deaths [4, 5, 21, 22].

A wide range of factors might influence healthcare professionals’ behaviours, decision-making and clinical practice [23]. Theory-driven approaches are often used to understand and predict clinicians’ behaviour in healthcare [24]. The social exchange theory has been used to explain physician referral behaviour in various healthcare settings [25–29]. However, its usefulness in a cancer palliative care setting is not known. In this study, we explored the utility of social exchange theory as an explanatory framework to describe oncologists’ palliative care referral behaviour to hospital-based specialist palliative care teams in a cancer care setting.

## Methods

Our hypothesis was that social exchange theory is a helpful framework for explaining oncologists’ palliative care referral behaviour in a cancer setting, and the hypothetico-deductive method was used to test this contention. [30]. The hypothetico-deductive approach enables the exploration of how data contributes to testing a hypothesis and its role in confirming or undermining it [30]. Furthermore, it formalises and demonstrates the relationship between the hypothesis and the data [30]. In a hypothetico-deductive method, an established theoretical framework is tested against the data collected through observations [31]. Using empirical evidence allows for testing, modifying, and rejecting a theoretical framework [32]. The premise of Karl Popper’s scientific philosophy is that all dogmas and theories should be tested and cautioned against their uncritical adoption [33]. Furthermore, the researchers are confronted with data that needs explanation. Testable hypotheses are deduced from existing theories, and scientific data can be used to test a hypothesis. Confirmed hypotheses are kept or modified, and falsified ones are rejected [34]. Therefore, we chose Popper’s hypothetico-deductive method for our scientific inquiry. Karl Popper’s hypothetico-deductive approach has seven steps, detailed in the next section [35].

## Results

### Step 1: Identifying a broad problem area

#### Broad problem area: Timely palliative care referral in a cancer setting

Research has shown that both adults and children benefit from timely referral. It improved quality of life, enhanced symptom control, bettered communication, informed treatment decision-making, advance care planning, end-of-life care, and reduced costs [36–41]. In a cancer care setting, oncologists may act as gatekeepers, and their gatekeeping behaviour can either help or impede referrals to palliative care [42, 43]. Studies have indicated that gatekeeping behaviours may involve delaying a referral for palliative care until the end of a potentially curative treatment or only making a referral when explicitly requested by the patient’s family [44–46]. Additionally, it has been revealed that oncologists prefer controlling and coordinating the care process of their patients at every stage of their illness trajectories, including the referral process [47–52]. Therefore, it’s essential to comprehend oncologists’ referral behaviour in a cancer setting to improve engagement and early integration of services.

### Step 2: Defining a problem statement

#### Problem statement: Referral behaviour of oncologists underpins timely palliative care referrals

This research focused on the significance of timely referrals for palliative care and how oncologists’ behaviour affects this process. It considers the social and cultural context and all stakeholders’ viewpoints on healthcare referrals [43]. Understanding their perspectives on what promotes or impedes referrals can inform changes in policies and practices that have the potential to address health disparities and bring about transformative improvements [53]. In some healthcare settings, social exchange theory has been used before to explain how physicians make referrals [25–29]. These studies showed a strong correlation between referral exchange behaviour and the principles of social exchange theory, which is described in Table 1. However, none of these studies was conducted in a cancer care setting. In this study, we tested the utility of social exchange theory as a framework for explaining oncologists’ referral behaviour in a cancer palliative care setting using a systematic review [54] and original research [55] conducted by the authors of this paper.

**Table 1:** Components of Social Exchange Theory

Sl. No	Component	Description
1	Sentiment	Sentiments are the views and attitudes of one social actor towards another involved in the exchange [57]. Presuppositions, power and motivation are the common social exchange sentiments [58]. Presuppositions are a preformed notion about the exchange or the persons involved in the exchange based on previous experiences, knowledge or biases [58]. Power in social exchange is linked to the social actor’s virtues in the society or situation and depends upon another person’s dependency on the social actor [59]. Therefore, it creates a power dependency relationship [59]. Motivation is the desire of the social actor to achieve something of value that is closely associated with the satisfaction of achieving it [60].
2	Task	The task corresponds to the effort required to achieve an activity. Structural and contextual conditions determine the efforts needed to accomplish the task [61]. Moreover, emotions triggered by the task activities also determine the person’s involvement in the task [57]. Therefore, the jointness of exchange does not only depends upon the reward of the exchange but also on the structural and contextual conditions constraining or enabling the task and the emotions triggered by it [57].
3	Interaction	Interaction relates to the process of exchange [58]. There are three kinds of exchange. The trade exchange is purely in terms of economic gains and losses, and the power exchange is hierarchical, where there is limited choice and freedom to the persons participating in the exchange [58]. As the act of referral is a social behaviour [29], in this research, I am focusing on the behavioural exchange, where the relationships are voluntary and interdependent, and behaviours act as tendencies in the exchange process [60]. The person participating in the exchange takes into account the task, reward and cost of interacting and will continue to interact if the interaction is in alignment with the person’s expected outcomes [60].
4	Reward	The rewards are the benefits of exchange. In the social exchange, rewards are discussed in terms of a person’s gains and its influence on the exchange process [62, 63]. There are several dimensions to the reward. Immediate rewards are based on the behavioural choices associated with a single event or an outcome. In contrast, long-term rewards are relational rewards based on the long-term association of social actors and cumulative outcomes of interaction [60]. Anticipatory rewards are the potential future rewards expected by the person in the exchange process [60]. Rewards are a form of reinforcement, and social relationships cease to exist unless the exchange reinforces the person’s expectations [56].
5	Cost	Cost in social exchange corresponds to the negative effects or losses sustained during achieving the reward [59, 62]. When costs involved in achieving the reward are high, then there is less chance of a person taking up the task [59]. The person’s decision to choose an activity, forego it or choose an alternative depends upon the costs associated with it [59].
6	Profit	Profit relates to a person’s expectation of rewards and the costs incurred in the process. Moreover, in a social exchange, the proportion of rewards to the costs should be a fair process [59, 62].
7	Value	Value means not just the assessment of profit due to interaction but the feelings of satisfaction achieved by doing that activity [58]. The value associated with doing an activity and the sentiments the person gets from another person during the process of activity determines the long-term association with the activity [58]. The person continues to do the activity even if the activity does not bring profit if it provides satisfaction and adds value [60].
8	Comparison Level	Comparison level is the standard against which the social actor measures the utility of the rewards [64]. The standard could be based on the previous or current experience of rewards and alternate choices that are available [64]. This perception is based not just on the magnitude of immediate rewards but also on the cumulative slope of the reward [58]. Therefore, one activity or an outcome of a single palliative referral is unlikely to make a person choose an alternative or influence the referral behaviour [58]. If the cumulative perception is negative and an alternative is more profitable, then the person may choose to terminate the relationship and choose the alternative [58, 64].

### Step 3: Develop a hypothesis

#### Hypothesis: Social exchange theory is useful for explaining oncologists’ palliative care referral behaviour in a cancer setting

Referring patients in a healthcare setting is a form of social interaction that involves sharing responsibility for patient care between the referrer and referee, which can be explained through social exchange behaviour [28, 29]. Social exchange theory explains how people’s social behaviour is influenced by the possibility of gaining or losing something of value through an exchange [56]. Social interactions are usually seen as a means for individuals to fulfil their needs, seek rewards and avoid costs [56]. The components included in the social exchange process are detailed in Table 1.

### Steps 4,5 and 6: Determining measures for hypothesis testing, data collection and analysis

#### Measures for hypothesis testing, data collection and analysis

The hypothesis was tested using a systematic review [54] and original research [55] conducted by the authors of this paper. The data collection and analysis of these two measures are described below.

We conducted a systematic review [54] to answer the question: “What do oncologists and haematologists think about referring patients to palliative care?” We looked at studies published in English that involved human subjects from January 1, 1990, to December 31, 2019. The studies we included focused on the opinions of oncologists, haematologists, and cancer specialists regarding referring patients to palliative care in a cancer care setting. To ensure review quality, we assessed the methodological rigour of all studies using Hawker’s tool [57]. Only studies with a score of 19 or higher were included. Two reviewers independently conducted screening, quality appraisal, and data extraction. This review used various evidence, including surveys and qualitative and mixed-method studies. We chose Popay’s narrative synthesis to analyse study findings, ideal for identifying common themes from textual data gathered through surveys and qualitative studies [58]. Furthermore, Popay’s method enables using a theoretical framework for interpreting study findings [58]. After reviewing a database of 9336 citations, we found 23 relevant studies for our synthesis. Through this process, we developed five themes related to presuppositions held by oncologists and haematologists, power dynamics and trust issues, challenges in making a palliative care referral, weighing the costs and benefits of a referral, and strategies to facilitate the referral process [54].

A qualitative study [55] was conducted to study the views of cancer specialists on aspects that either support or impede palliative care referral in paediatric oncology. We recruited 22 oncologists and haematologists who manage children with cancer from 13 tertiary cancer centres. We chose these centres based on three criteria: they offer paediatric oncology and haematology services, they have oncologists and haematologists who manage children with cancer, and they provide palliative care services. We gathered research data through individual face-to-face semi-structured qualitative interviews and analysed the data using Braun and Clarke’s Reflexive Thematic Analysis method [59]. Through data analysis, we have generated four key themes: attitudes and ideas regarding palliative care and referrals, the steps involved in referring a patient to palliative care, assessing the advantages and disadvantages of referral, and creating successful approaches for incorporating palliative care into paediatric oncology [55].

### Step 7.1: Data interpretation and theory evaluation

*The findings of the systematic review* [54] *and the original research* [55] *were used to test the hypothesis; the data was interpreted using the social exchange theory.*

#### Human cognition and emotions

Human cognition is an essential motivation for social exchange that goes beyond the process or outcome of the exchange [60]. The social actors are emotive and cognising, and the emotions experienced by the social actors act as an internal reinforcement for the exchange behaviour [61]. However, sentiments go beyond emotions, representing an affective state or feeling where emotions are linked to a social object or social unit [62]. It is a social construct that leads to an affective response, which is the psychological state of the social actor [63]. The presupposition is an implicit assumption or belief about a phenomenon [64]. Presuppositions trigger cognitive responses that impact decision-making and social behaviour [65].

Oncologists hold certain beliefs about the reliability of palliative care providers, as shown in both review and research studies [54, 55]. Trust is a cognitive process that involves one person expecting another to be trustworthy [66]. Trustworthiness is believing in someone’s ability, reliability, integrity, resourcefulness, and benevolence [67]. In both review and research findings, oncologists emphasised the importance of competence-based trust when referring patients to another person or team for effective task performance [68].

The study [55] brought out benevolence as a facet of trustworthiness. Being benevolent means doing good and being kind. [69]. In the study [55], oncologists felt that some palliative care providers were less benevolent due to a perceived lack of empathy and a lacklustre approach. The study [55] noted that social actors’ identities impact trustworthiness, cognition, and exchange behaviour [62].

In the review [54], oncologists reported feeling confident in their ability to provide care. However, the study [55] found that many oncologists had mixed feelings about their ability to respond to these needs appropriately. Perceived self-efficacy refers to a person’s belief in their ability to perform a task to meet their and others’ expectations [70]. This belief impacts cognitive and emotional processes, social behaviour and actions [70]. The study [55] found that only a few oncologists felt confident providing palliative care. Most acknowledged the benefits of referring patients to palliative care but recognised their limitations in providing these services due to a lack of skills and knowledge. This awareness of their limitations and self-efficacy influenced their referral behaviours.

Emotions triggered by task activities are central to social exchange behaviour [61]. The exchange process can trigger a host of emotions. Some are general feelings like pleasure or dissatisfaction, while others are specific feelings like anger, shame, trust, confidence, gratitude or pride [71]. The exchange outcomes also produce emotions that influence the social actor’s commitment to the exchange process [71]. A positive emotion triggered will encourage the social actor to repeat the experience, whereas a negative emotion may deter future participation in the exchange process [71].

The review found that [54] some oncologists experienced negative emotions such as therapeutic failure, abandonment, and a break in the therapeutic relationship when referring patients to palliative care. It could also lead to a loss of hope and hinder future engagement. On the other hand, the study [55] found that positive feedback from families about the quality of palliative care services was seen as a reinforcement for future referrals by oncologists.

#### Power, status and expectations

In social exchange, the social actor making the referral retains the reward power, whereas another social actor is rewarded with the referral if they meet the expectations of the referrer [72]. In the study [55], oncologists made a referral to palliative care if those services met referrer expectations and agreed with the line of management advised by referring oncologists. This one-sided dependency leads to asymmetrical relationships where the person receiving the referral must comply with the person’s wishes for making the referral [72]. and can lead to coercive power, in which a social actor obtains compliance from another [72]. This phenomenon was observed in our review [54] and research [55] findings. The oncologists wanted to oversee and manage the patient’s care throughout the illness, even when palliative care providers were involved. They desired to maintain control over the patient. It is an example of a social actor using their status or superior attributes to command compliance. [73].

Expert power is where the social actor believes they have expert skills and knowledge in a domain not possessed by another social actor, and legitimate power is derived from the virtue of their position [74]. According to our study [55], oncologists’ qualifications and experience influenced their decisions to refer patients. Due to their training, oncologists believed they were more qualified and experienced than palliative care providers. They also felt they had the authority to control and coordinate all referral activities [63]. The review supported the study findings [54], which showed that oncologists had the power to control the referral process and saw palliative care referral as interference in their care process, leading them to gatekeep the process.

#### Symbolism and stigma

Exchange behaviour also has a symbolic perspective where social actors interact and communicate about a phenomenon using symbolic inferences [63]. In the review [54], oncologists believed that referring patients to palliative care signified a loss of hope, a disconnection in the therapeutic relationship, and abandonment. In the study [55], oncologists compared the relationship between themselves and their patients to that of a family. They saw palliative care referral as equivalent to handing a family member to someone else, indicating a failure in treatment and letting down the patient. Oncologists also observed that families saw palliative care referral as an indication of a change in the patient’s condition, a shift in treatment goals, or the possibility that the patient may not recover.

Stigma is a complex phenomenon characterised by stereotypes, prejudices and discrimination [75]. In the study [55] and review [54] findings, Oncologists hesitated to recommend palliative care due to its negative association with death. Public stigma refers to stereotyped thoughts based on general opinion [76]. Oncologists felt that this public stigma leads families to avoid considering palliative care. According to the study [55], both families and oncologists have unfavourable views of palliative care, with oncologists avoiding the term altogether as it can induce fear and symbolise a loss of hope. Label avoidance stigma occurs when someone avoids a particular management strategy because of the negative connotations that come with its name [77]. In the review [54] and the study [55], label avoidance stigma is also an issue as some oncologists avoid the term palliative care altogether, as it requires them to explain the concept to families. Furthermore, the perception of public stigma and label avoidance stigma can give oncologists the power to exclude palliative care providers from the care process [78].

#### Task and efforts

The effort needed to complete a task impacts future exchange [62]. The contribution of a social actor towards the task is influenced by how fairly the effort-to-reward ratio is perceived [79]. Should this balance be unequal, the social actor’s interest in the exchange relationship may decrease [80]. Consequently, the social actor’s perception of the effort-to-reward ratio moderates social exchange behaviour [81].

In both review [54] and the study [55], oncologists found it challenging to make a referral for palliative care due to the many illness-related factors they had to consider. It includes aspects like progression of the disease, any complications, the stage of the illness, the presence of symptoms, the potential for a cure, the intent of treatment, the patient’s prognosis, and their performance status. It was a significant effort to navigate this complex set of factors [54, 55].

#### Reward, cost, profit and value

Reinforcement is the act of selectively repeating a behaviour [56]. In social exchange relationships, reinforcement is a crucial concept, as it is closely tied to rewards, costs, profits, and value [82]. Socially significant actions will not be repeated unless reinforced [82]. Various rewards are discussed from a social exchange perspective [83]. These are not limited to physiological or materialistic benefits but can help fulfil higher self-actualisation needs [83].

The rewards from certain behaviours are connected to the immediate outcomes of those actions, which can impact future interactions [83]. Social actors first notice these rewards because they result from short-term associations [83]. In the review [54] and the study [55], oncologists appreciated behavioural rewards such as pain and symptom management, improved quality of life, better family coping, support for decision-making and advance care planning. The study [55] found that they appreciated the support provided by palliative care services for children at home during the terminal phase of the illness.

The benefits of having a continuous and extended relationship with others are known as relational rewards [83]. However, sometimes these rewards may not be immediately apparent due to the long-term nature of the relationship [83]. Some oncologists participating in the study [55] believed that a collaborative relationship could improve oncologists’ productivity, reduce stress, improve treatment outcomes, and share responsibility for care. Saving the oncologists’ time was the only relational reward noted in the review [54]. Self-actualisation rewards can bring about personal growth [83]. Self-actualisation rewards were only seen in the study findings [55]. A few oncologists reported that their association with palliative care had improved their symptom management and prognostication skills and their ability to empathise and show compassion.

When engaging in social exchange, the cost refers to the loss sustained in pursuing rewards [84]. It can be seen as either removing a positive reinforcer or applying a negative reinforcer [63]. If the cost is too high, it may cause the social actor to opt out of the exchange or choose an alternative [63]. The frequency of an activity is directly related to its cost, with more costly activities being undertaken less often. Profit, on the other hand, is the reward minus the cost. As long as the social actor stands to gain from the exchange, the process may continue [63]. The review [54] and the study [55] found that palliative care referrals can sometimes lead to confusion and mixed messages for the patient’s family. It occurs when the palliative care team provides conflicting information about the patient’s clinical condition, prognosis, and outcomes, leading to a disadvantage for the oncologists.

In addition to considering rewards, costs, and profits, social actors also consider the value of exchange [83]. This value is determined by the satisfaction the social actor derives from the activity and the positive emotions experienced during the exchange with another social actor [63]. Beyond the immediate and relational rewards, in the study [55], oncologists felt that early palliative care provides value to both patients and their families, as it helps build rapport with the palliative care team, ensures smooth transitions of care, and allows for symptom control and supportive care during cancer treatment. Additionally, it benefits oncologists by providing a reliable partner in the care process with whom they can trust, work with, and share responsibilities. The reciprocity norm dictates that the benefit received should be returned, and the provider should not be harmed [63]. Oncologists participating in the study [55] felt that palliative care providers should feel valued. According to the oncologists, palliative care providers should be valued and included as part of the oncology team to improve family acceptance of palliative care [55]. These align with earlier findings corresponding to power relationships and cost. By inviting palliative care providers to be part of the oncology team, oncologists can avoid sending mixed messages to patients and their families, which they identified as a cost of palliative care referral.

## Discussion

Social exchange theory [56] was initially only used to explain economic transactions in business relationships [85] and has faced criticism for having overlapping concepts and inadequate characterisation of domains and for portraying exchanges as purely economic [86, 87]. Despite this, the theory has since been applied to describe various human relationships outside economic contexts [88–91]. Social exchange theory has been previously used to understand physician referral behaviour in different clinical settings [25–29]. This is the first paper studying the utility of social exchange theory in a cancer setting by exploring oncologists’ palliative care referral behaviour.

We aimed to determine if social exchange theory could explain oncologists’ referral behaviour for palliative care [56]. Using a theoretical framework can assist in better comprehending and interpreting study or review results [92]. Evaluating a theory based on the data is essential, and empirical findings from a systematic review or study may either support, reject, or adjust the theory with an explanation [93]. We assessed the social exchange theory’s relevance to cancer palliative care using a systematic review [54] and original research [55].

### Step 7.2. Theory revision underpinned by data analysis

In this step the social exchange theory was evaluated in the light of empiric findings and modifications to theory was suggested. The first three study themes [55] and the initial four review themes [54] mentioned before fit well with social exchange theory [56]. However, when it came to discussing the strategies provided by oncologists to improve palliative care integration, we felt that social exchange theory was insufficient. As a result, we would like to suggest a critique and modification to the theory [56].

According to the social exchange theory, there are two levels of appraisal: the comparison level and the comparison level for alternatives [94, 95]. The comparison level evaluates the benefits and costs of social exchange, while the comparison level for alternatives involves considering other potential relationships that may be more rewarding [94, 95]. Social actors determine their level of satisfaction with a relationship based on the comparison level, and they remain committed to it as long as it is more profitable than other alternatives [63]. This decision depends on their knowledge of other relationships and the potential rewards and costs associated with them [94].

**Fig. 1:**
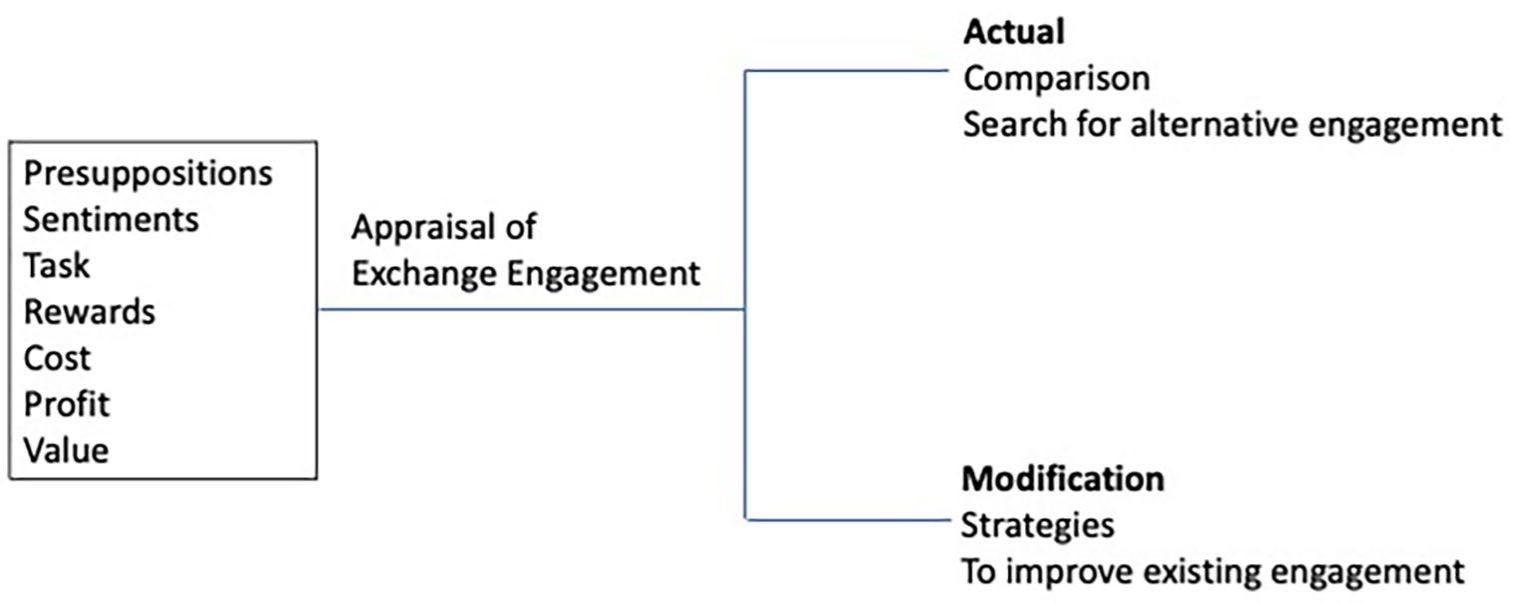
Suggested modification to the social exchange theory

In the systematic review [54] and study findings [55], oncologists appraised the exchange relationship regarding benefit and cost. However, they did not discuss the possibility of choosing an alternative approach to referral or palliative care services for their patients. Instead, they provided strategies through which relationships between oncology and palliative care teams can be fostered and bettered, and the exchange process of referral may be improved. Sometimes, people may continue with a relationship due to a lack of better options or dependence [96, 97]. However, in both the systematic review [54] and study findings [55], the oncologists valued the benefits of the relationship and provided strategies to integrate the teams. Therefore, it is suggested that adding feedback and solutions alongside a comparison level for alternatives represents a modification to the social exchange theory resulting from the systematic review data [54] and original research [55] (See Fig. 1).

### Limitation and strengths

In the systematic review [54], The review analysed different types of studies, such as surveys, qualitative studies, and mixed methods studies, and found similar results. Furthermore, studies included in the review had participants from various oncology backgrounds and locations. The systematic review used to test social exchange theory had 21 primary studies from adult oncology and only two from paediatric oncology. Moreover, the data from the original study [55] used for theory testing had only paediatric oncologists as the participants. Therefore, the generalisability of findings to either adult or paediatric settings might be challenging, which could be a limitation. Furthermore, the systematic review [54] and the original research [55] focused on referral behaviour from the oncologists’ perspective but did not consider the views of family members, palliative care providers, or other organizational factors.

## Conclusion

In a cancer care setting, oncologists play a role in determining whether or not a patient is referred to palliative care. Social exchange theory has been used to explain physician referral behaviour in various healthcare settings other than cancer. Popper’s hypothetico-deductive method demonstrated the utility of social exchange theory as an explanatory framework to describe oncologists’ palliative care referral behaviour in a cancer setting. Most of the findings of the systematic review [54] and the original research [55] used to test the hypothesis correlated well with the tenets of social exchange theory. However, social exchange theory was limited by its lack of utility in interpreting study [55] and review [54] findings concerning strategies provided by the oncologists to enhance palliative care integration. Therefore, a theory revision is suggested by adding feedback and solutions alongside a comparison level for alternatives representing a modification to the social exchange theory resulting from the data used to test the hypothesis.

## Data Availability

The data produced or examined during this research has been incorporated into the published article.

## References

[1] Fraser LK, Miller M, McKinney PA, Parslow RC, Feltbower RG. Referral to a specialist paediatric palliative care service in oncology patients. Pediatr Blood Cancer. 2011;56(4):677–80. 21298761 10.1002/pbc.22667

[2] Johnston DL, Vadeboncoeur C. Palliative care consultation in pediatric oncology. Support Care Cancer. 2012;20(4):799–803. 21479523 10.1007/s00520-011-1152-6

[3] Levine DR, Johnson LM, Snyder A, Wiser RK, Gibson D, Kane JR, et al. Integrating palliative care in pediatric oncology: evidence for an evolving paradigm for comprehensive cancer care. JNCCN J Natl Compr Cancer Netw. 2016;14(6):741–8. 10.6004/jnccn.2016.0076 PMC5357562 27283167

[4] Menon BS, Mohamed M, Juraida E, Ibrahim H. Pediatric cancer deaths: curative or palliative? J Palliat Med. 2008;11(10):1301–3. 19115887 10.1089/jpm.2008.0167

[5] Tzuh Tang S, Hung YN, Liu TW, Lin DT, Chen YC, Wu SC, et al. Pediatric end-of-life care for Taiwanese children who died as a result of cancer from 2001 through 2006. J Clin Oncol. 2011;29(7):890–4. 21189377 10.1200/JCO.2010.32.5639

[6] Ullrich CK, Lehmann L, London WB, Guo D, Sridharan M, Koch R, et al. End-of-life care patterns Associated with Pediatric Palliative Care among children who underwent hematopoietic stem cell transplant. Biol Blood Marrow Transplant. 2016;22(6):1049–55. 26903381 10.1016/j.bbmt.2016.02.012 PMC5541943

[7] Adusumilli P, Nayak L, Viswanath V, Digumarti L, Digumarti RR. Palliative care and end-of-life measure outcomes: experience of a tertiary care institute from South India. South Asian J Cancer. 2018;7(3):210–3. 30112344 10.4103/sajc.sajc_257_17 PMC6069332

[8] Atreya S. Referral patterns of Gynecological Cancer patients to a Palliative Medicine Unit: a 2 years retrospective analysis. Indian J Palliat Care. 2017;23(4):409–12. 29123347 10.4103/IJPC.IJPC_77_17 PMC5661343

[9] Chatterjee A, Nimje G, Jain PN. Referral pattern to a Tertiary Care Cancer Pain Clinic in India. J Pain Palliat Care Pharm. 2019;33(1–2):6–14. 10.1080/15360288.2019.1631240 31260382

[10] Ghoshal A, Salins N, Damani A, Deodhar J, Muckaden M. Specialist pediatric palliative care referral practices in pediatric oncology: a large 5-year retrospective audit. Indian J Palliat Care. 2016;22(3):266–73. 27559254 10.4103/0973-1075.185031 PMC4973486

[11] Sharma K, Mohanti BK, Rath GK, Bhatnagar S. Pattern of palliative care, pain management and referral trends in patients receiving radiotherapy at a tertiary cancer center. Indian J Palliat Care. 2009;15(2):148–54. 20668595 10.4103/0973-1075.58462 PMC2902117

[12] Sinha S, Matharu JK, Jacob J, Palat G, Brun E, Wiebe T, et al. Cancer Treatment and End-of-Life Care. J Palliat Med. 2018;21(8):1100–6. 29768074 10.1089/jpm.2017.0695

[13] Feudtner C, Kang TI, Hexem KR, Friedrichsdorf SJ, Osenga K, Siden H, et al. Pediatric palliative care patients: a prospective multicenter cohort study. Pediatrics. 2011;127(6):1094–101. 21555495 10.1542/peds.2010-3225

[14] Jalmsell L, Kreicbergs U, Onelöv E, Steineck G, Henter J-I. Symptoms affecting children with malignancies during the last month of life: a nationwide follow-up. Pediatrics. 2006;117(4):1314–20. 16585329 10.1542/peds.2005-1479

[15] Jordan RI, Allsop MJ, ElMokhallalati Y, Jackson CE, Edwards HL, Chapman EJ et al. Duration of palliative care before death in international routine practice: a systematic review and meta-analysis. BMC Med. 2020;18(1). 10.1186/s12916-020-01829-x PMC7690105 33239021

[16] Clark D, Centeno C, Clelland D, Garralda E, López-Fidalgo J, Downing J, et al. In: Connor SR, editor. How are palliative care services developing worldwide to address the unmet need for care? London, UK: Worldwide Hospice Palliative Care Alliance; 2020.

[17] Downing J, Boucher S, Daniels A, Nkosi B. Paediatric Palliative Care in Resource-Poor Countries. Child (Basel Switzerland). 2018;5(2):27. 10.3390/children5020027 PMC5835996 29463065

[18] Rost M, Acheson E, Kühne T, Ansari M, Pacurari N, Brazzola P, et al. Palliative care in Swiss pediatric oncology settings: a retrospective analysis of medical records. Support Care Cancer. 2018;26(8):2707–15. 29478188 10.1007/s00520-018-4100-x

[19] Bradshaw G, Hinds PS, Lensing S, Gattuso JS, Razzouk BI. Cancer-related deaths in children and adolescents. J Palliat Med. 2005;8(1):86–95. 15662177 10.1089/jpm.2005.8.86

[20] De Graves S, Aranda S. Exploring documentation of end-of-life care of children with cancer. Int J Palliat Nurs. 2002;8(9):435–43. 12362125 10.12968/ijpn.2002.8.9.10688

[21] Howell DA, Shellens R, Roman E, Garry AC, Patmore R, Howard MR. Haematological malignancy: are patients appropriately referred for specialist palliative and hospice care? A systematic review and meta-analysis of published data. Palliat Med. 2011;25(6):630–41. 21228094 10.1177/0269216310391692

[22] Fowler K, Poehling K, Billheimer D, Hamilton R, Wu H, Mulder J, et al. Hospice referral practices for children with cancer: a survey of pediatric oncologists. J Clin Oncol. 2006;24(7):1099–104. 16505429 10.1200/JCO.2005.02.6591

[23] Godin G, Bélanger-Gravel A, Eccles M, Grimshaw J. Healthcare professionals’ intentions and behaviours: a systematic review of studies based on social cognitive theories. Implement Sci. 2008;3(1):36. 18631386 10.1186/1748-5908-3-36 PMC2507717

[24] Matthew B, Perkins MD, Peter MBA, Jensen S, James Jaccard MD, Peter Gollwitzer PD, Gabriele Oettingen PD, Elizabeth Pappadopulos PD. Applying theory-driven approaches to understanding and modifying clinicians’ behavior: what do we know? Psychiatric Serv. 2007;58(3):342–8. 10.1176/ps.2007.58.3.342 17325107

[25] Byrd ME. Social exchange as a framework for client-nurse interaction during public health nursing maternal-child home visits. Public Health Nurs. 2006;23(3):271–6. 16684206 10.1111/j.1525-1446.2006.230310.x

[26] Grembowski DE, Cook K, Patrick DL, Roussel AE. Managed care and physician referral. Med Care Res Rev. 1998;55(1):3–31. 9529879 10.1177/107755879805500101

[27] Lamb GS. Two explanations of nurse practitioner interactions and participatory decision making with physicians. Res Nurs Health. 1991;14(5):379–86. 1909808 10.1002/nur.4770140509

[28] Prizer LP, Gay JL, Perkins MM, Wilson MG, Emerson KG, Glass AP, et al. Using social exchange theory to understand non-terminal palliative care referral practices for Parkinson’s disease patients. Palliat Med. 2017;31(9):861–7. 28659011 10.1177/0269216317701383

[29] Shortell SM. Determinants of physician referral rates: an exchange theory approach. Med Care. 1974;12(1):13–31. 4811399 10.1097/00005650-197401000-00002

[30] Sprenger J. Hypothetico-deductive confirmation. Philos Compass. 2011;6(7):497–508.

[31] Tariq MU. Hypothetico-deductive method: a comparative analysis. JOBARI. 2015;7:228–31.

[32] Fardet A, Lebredonchel L, Rock E. Empirico-inductive and/or hypothetico-deductive methods in food science and nutrition research: which one to favor for a better global health? Crit Rev Food Sci Nutr. 2021:1–14. 10.1080/10408398.2021.1976101 34494476

[33] Popper KR. What is Dialectic? Mind. 1940;49(196):403–26.

[34] Mahootian F, Eastman TE. Complementary frameworks of Scientific Inquiry: Hypothetico-Deductive, Hypothetico-Inductive, and observational-inductive. World Futures. 2009;65(1):61–75.

[35] Martini C. Hypothetico-Deductive Method. The Wiley‐Blackwell Encyclopedia of Social Theory. 2017:1–3.

[36] Brumley R, Enguidanos S, Jamison P, Seitz R, Morgenstern N, Saito S, et al. Increased satisfaction with care and lower costs: results of a randomized trial of in-home palliative care. J Am Geriatr Soc. 2007;55(7):993–1000. 17608870 10.1111/j.1532-5415.2007.01234.x

[37] Greer JA, Pirl WF, Jackson VA, Muzikansky A, Lennes IT, Heist RS, et al. Effect of early palliative care on chemotherapy use and end-of-life care in patients with metastatic non-small-cell lung cancer. J Clin Oncol. 2012;30(4):394–400. 22203758 10.1200/JCO.2011.35.7996

[38] Mitchell S, Slowther A-M, Coad J, Bertaud S, Dale J. Facilitators and barriers to the delivery of palliative care to children with life-limiting and life-threatening conditions: a qualitative study of the experiences and perceptions of healthcare professionals. Arch Dis Child. 2021:archdischild–2021. 10.1136/archdischild-2021-321808 33980510

[39] Temel JS, Greer JA, Muzikansky A, Gallagher ER, Admane S, Jackson VA, et al. Early palliative care for patients with metastatic non-small-cell lung cancer. N Engl J Med. 2010;363(8):733–42. 20818875 10.1056/NEJMoa1000678

[40] Weaver MS, Rosenberg AR, Tager J, Wichman CS, Wiener L. A Summary of Pediatric Palliative Care Team Structure and Services as reported by centers Caring for Children with Cancer. J Palliat Med. 2018;21(4):452–62. 29173030 10.1089/jpm.2017.0405 PMC5915222

[41] Zernikow B, Szybalski K, Hübner-Möhler B, Wager J, Paulussen M, Lassay L, et al. Specialized pediatric palliative care services for children dying from cancer: a repeated cohort study on the developments of symptom management and quality of care over a 10-year period. Palliat Med. 2019;33(3):381–91. 30537890 10.1177/0269216318818022

[42] Dalberg T, McNinch NL, Friebert S. Perceptions of barriers and facilitators to early integration of pediatric palliative care: a national survey of pediatric oncology providers. Pediatr Blood Cancer. 2018;65(6):e26996. 29418063 10.1002/pbc.26996

[43] Kars MC, van Thiel GJ, van der Graaf R, Moors M, de Graeff A, van Delden JJ. A systematic review of reasons for gatekeeping in palliative care research. Palliat Med. 2016;30(6):533–48. 26577927 10.1177/0269216315616759

[44] Nyirő J, Zörgő S, Enikő F, Hegedűs K, Hauser P. The timing and circumstances of the implementation of pediatric palliative care in Hungarian pediatric oncology. Eur J Pediatr. 2018;177(8):1173–9. 29785662 10.1007/s00431-018-3170-6

[45] Prod’homme C, Jacquemin D, Touzet L, Aubry R, Daneault S, Knoops L. Barriers to end-of-life discussions among hematologists: a qualitative study. Palliat Med. 2018;32(5):1021–9. 29756557 10.1177/0269216318759862

[46] Sarradon-Eck A, Besle S, Troian J, Capodano G, Mancini J. Understanding the barriers to Introducing Early Palliative Care for patients with Advanced Cancer: a qualitative study. J Palliat Med. 2019;22(5):508–16. 30632886 10.1089/jpm.2018.0338

[47] Cherny NI, Catane R. Attitudes of medical oncologists toward palliative care for patients with advanced and incurable cancer: report on a survery by the European Society of Medical Oncology Taskforce on Palliative and supportive care. Cancer. 2003;98(11):2502–10. 14635087 10.1002/cncr.11815

[48] Hay CM, Lefkowits C, Crowley-Matoka M, Bakitas MA, Clark LH, Duska LR, et al. Gynecologic oncologist views influencing referral to Outpatient Specialty Palliative Care. Int J Gynecol Cancer. 2017;27(3):588–96. 28060140 10.1097/IGC.0000000000000893 PMC5315630

[49] Horlait M, Chambaere K, Pardon K, Deliens L, Van Belle S. What are the barriers faced by medical oncologists in initiating discussion of palliative care? A qualitative study in Flanders, Belgium. Support Care Cancer. 2016;24(9):3873–81. 27086311 10.1007/s00520-016-3211-5

[50] Rhondali W, Burt S, Wittenberg-Lyles E, Bruera E, Dalal S. Medical oncologists’ perception of palliative care programs and the impact of name change to supportive care on communication with patients during the referral process. A qualitative study. Palliat Support Care. 2013;11(5):397–404. 23302500 10.1017/S1478951512000685

[51] Schenker Y, Crowley-Matoka M, Dohan D, Rabow MW, Smith CB, White DB, et al. Oncologist factors that influence referrals to subspecialty palliative care clinics. J Oncol Pract. 2014;10(2):e37–44. 24301842 10.1200/JOP.2013.001130 PMC3948709

[52] Wright B, Forbes K. Haematologists’ perceptions of palliative care and specialist palliative care referral: a qualitative study. BMJ Support Palliat Care. 2017;7(1):39–45. 25252939 10.1136/bmjspcare-2014-000689

[53] McGorty EK, Bornstein BH. Barriers to physicians’ decisions to discuss hospice: insights gained from the United States hospice model. J Eval Clin Pract. 2003;9(3):363–72. 12895158 10.1046/j.1365-2753.2003.00406.x

[54] Salins N, Ghoshal A, Hughes S, Preston N. How views of oncologists and haematologists impacts palliative care referral: a systematic review. BMC Palliat Care. 2020;19(1):175. 33228651 10.1186/s12904-020-00671-5 PMC7686696

[55] Salins N, Hughes S, Preston N. Presuppositions, cost-benefit, collaboration, and competency impacts palliative care referral in paediatric oncology: a qualitative study. BMC Palliat Care. 2022;21(1):215. 36456939 10.1186/s12904-022-01105-0 PMC9717409

[56] Ekeh PP. Social exchange theory: The two traditions: Heinemann London; 1974. Pages 127–132 p.

[57] Hawker S, Payne S, Kerr C, Hardey M, Powell J. Appraising the evidence: reviewing disparate data systematically. Qual Health Res. 2002;12(9):1284–99. 12448672 10.1177/1049732302238251

[58] Popay J, Roberts H, Sowden A, Petticrew M, Arai L, Rodgers M et al. Guidance on the conduct of narrative synthesis in systematic reviews. A product from the ESRC methods programme version. 2006;1:b92.

[59] Braun V, Clarke V, Hayfield N, Terry G. Thematic analysis. In: Liamputtong P, editor. Handbook of Research Methods in Health Social Sciences. Singapore: Springer Singapore; 2019. pp. 843–60.

[60] Poonamallee L, Goltz S, editors. Beyond Social Exchange Theory: An Integrative Look at Transcendent Mental Models for Engagement 2012 p64.

[61] Lawler EJ, Thye SR, editors. Social Exchange Theory of Emotions 2006 p281-282.

[62] Lawler EJ. An affect theory of social exchange. Am J Sociol. 2001;107(2):321–52.

[63] Cook KS, Hahn M. Social exchange theory: current status and future directions. Theoretical Sociol. 2021:179–205.

[64] Simons M. On the conversational basis of some presuppositions. Semant Linguistic Theory. 2013;11:329–48.

[65] Domaneschi F, Carrea E, Penco C, Greco A. The cognitive load of presupposition triggers: mandatory and optional repairs in presupposition failure. Lang Cognition Neurosci. 2014;29:136–46.

[66] Nunkoo R, Ramkissoon H. Power, trust, social exchange and community support. Annals Tourism Res. 2012;39:997–1023.

[67] Kim S, Kuo M-H. Examining the relationships among Coaching, trustworthiness, and role behaviors: a Social Exchange Perspective. J Appl Behav Sci. 2015;51(2):152–76.

[68] Lee H. The role of competency based trust and organizational identification in continuous improvement. J Managerial Psychol. 2004;19:623–39.

[69] Colquitt J, Rodell JB. Justice, Trust, and trustworthiness: a longitudinal analysis integrating three theoretical perspectives. Acad Manag J. 2011;54:1183–206.

[70] Maddux JE. Self-efficacy. Interpersonal and intrapersonal expectancies: Routledge; 2016. pp. 41 – 6.

[71] Lawler EJ, Thye SR, BRINGING, EMOTIONS INTO SOCIAL EXCHANGE THEORY. Rev Sociol. 1999;25:217–44.

[72] Cook KS, Cheshire C, Rice ER, Nakagawa S. Social exchange theory. Handbook of social psychology. 2013:61–88.

[73] Emerson R. Social exchange theory, annual review of sociology. Annuals Reviews. 1976 p335.

[74] Yukl G, Falbe CM. Importance of different power sources in downward and lateral relations. J Appl Psychol. 1991;76:416–23.

[75] Major B, O brien L. The social psychology of stigma. Ann Rev Psychol. 2005;56:393–421. 15709941 10.1146/annurev.psych.56.091103.070137

[76] Vogel DL, Bitman RL, Hammer J, Wade N. Is stigma internalized? The longitudinal impact of public stigma on self-stigma. J Couns Psychol. 2013;60 2:311–6. 23421775 10.1037/a0031889

[77] Smith RA, Hipper TJ. Label management: investigating how confidants encourage the use of communication strategies to avoid stigmatization. Health Commun. 2010;25(5):410–22. 20677045 10.1080/10410236.2010.483335

[78] Link BG, Phelan J. Stigma power. Soc Sci Med. 2014;103:24–32. 24507908 10.1016/j.socscimed.2013.07.035 PMC4451051

[79] Cropanzano R, Anthony EL, Daniels SR, Hall AV. Social exchange theory: a critical review with theoretical remedies. Acad Manag Ann. 2017;11(1):479–516.

[80] Lin T-C, Huang C-C. Withholding effort in knowledge contribution: the role of social exchange and social cognitive on project teams. Inf Manag. 2010;47:188–96.

[81] Janssen O. Job demands, perceptions of efforî reward fairness and innovative work behaviour. J Occup Organizational Psychol. 2000;73:287–302.

[82] Burns T. A structural theory of Social Exchange. Acta Sociol. 1973;16(3):188–208.

[83] Stafford L. Social Exchange Theories: Calculating the Rewards and Costs of Personal Relationships. 2008. pp. 377 – 90.

[84] Homans GC. Social behavior as exchange. Am J Sociol. 1958;63(6):597–606.

[85] Lambe CJ, Wittmann CM, Spekman RE. Social Exchange Theory and Research on Business-to-business Relational Exchange. J Business-to-Business Mark. 2001;8(3):1–36.

[86] Davies PS. Logical reasoning and domain specificity - A critique of the social exchange theory of reasoning. Biology Philos. 1995;10(1):1–37.

[87] Adongo R, Kim S, Elliot S. Give and take: a social exchange perspective on festival stakeholder relations. Annals Tourism Res. 2019.

[88] Bishop JW, Scott KD. An examination of organizational and team commitment in a self-directed team environment. J Appl Psychol. 2000;85(3):439. 10900817 10.1037/0021-9010.85.3.439

[89] Bowling NA, Beehr TA, Swader WM. Giving and receiving social support at work: the roles of personality and reciprocity. J Vocat Behav. 2005;67(3):476–89.

[90] Nakonezny PA, Denton WH. Marital relationships: a Social Exchange Theory Perspective. Am J Family Therapy. 2008;36(5):402–12.

[91] Ward C, Berno T. Beyond social exchange theory: attitudes toward tourists. Annals Tourism Res. 2011;38(4):1556–69.

[92] Fletcher AJ. Applying critical realism in qualitative research: methodology meets method. Int J Soc Res Methodol. 2017;20(2):181–94.

[93] Zhang T, Critical Realism. A critical evaluation. Social Epistemology. 2023;37(1):15–29.

[94] Thibaut J, Kelley H. Social exchange theory. A first look at communication theory. 2008:196–205.

[95] Smith RH, Diener E, Garonzik R. The roles of outcome satisfaction and comparison alternatives in envy. Br J Soc Psychol. 1990;29(3):247–55.

[96] Gelles RJ. An exchange/social control theory. The dark side of families: Current family violence research. 1983:151 – 65.

[97] Sabatelli RM, Cecil-Pigo EF. Relational interdependence and commitment in marriage. J Marriage Fam. 1985:931–7.

