# Correction: Oncologists’ palliative care referral behaviour: testing utility of social exchange theory as an explanatory framework

**DOI:** 10.1186/s12904-024-01594-1

**Published:** 2024-11-15

**Authors:** Naveen Salins, Sean Hughes, Nancy Preston

**Affiliations:** 1 Department of Palliative Medicine and Supportive Care, Kasturba Medical College, Manipal Academy of Higher Education, Manipal, Karnataka 576104 India; 2 Division of Health Research, Health Innovation One, Sir John Fisher Drive, Lancaster University, Lancaster, LA1 4AT UK

**Correction: BMC Palliat Care 23, 183 (2024)**

10.1186/s12904-024-01517-0.

Following publication of the original article [1], the author wants to update the Acknowledgements and Funding sections by removing the data in bold.

**Old acknowledgements section**

We thank the Tata Trusts, Narotam Sekhsaria Foundation, **Hamied Foundation**,** and Cipla Foundation** for supporting this study. We also extend our thanks to faculty of Lancaster University and Kasturba Medical College, Manipal, for their invaluable assistance, both directly and indirectly, in our research.

**New acknowledgements section**

We thank the Tata Trusts and Narotam Sekhsaria Foundation for supporting this study. We also extend our thanks to faculty of Lancaster University and Kasturba Medical College, Manipal, for their invaluable assistance, both directly and indirectly, in our research.

**Old funding section**

Naveen Salins expresses gratitude for the support given by the Tata Trusts, Narotam Sekhsaria Foundation, **Hamied Foundation**,** and Cipla Foundation** in conducting this study. However, Nancy Preston and Sean Hughes received no funding for this research.

**New funding section**

Naveen Salins expresses gratitude for the support given by the Tata Trusts and Narotam Sekhsaria Foundation in conducting this study. However, Nancy Preston and Sean Hughes received no funding for this research.

The original article has been updated.
